# Clinical outcomes and structural integrity of C-shaped rotator cuff tears after arthroscopic repair: comparison with crescent-shaped tears

**DOI:** 10.1186/s13018-018-0863-5

**Published:** 2018-06-19

**Authors:** Wonyong Lee, Sung-Jae Kim, Chong-Hyuk Choi, Yun-Rak Choi, Yong-Min Chun

**Affiliations:** Department of Orthopaedic Surgery, Arthroscopy and Joint Research Institute, Severance Hospital, Yonsei University College of Medicine, CPO Box 8044, 50-1 Yonsei-ro, Seodaemun-gu, Seoul, 120-752 Korea

**Keywords:** Rotator cuff tear, Arthroscopic repair, Type, Re-tear, Stiffness

## Abstract

**Background:**

We aimed to describe a new C-shaped tear configuration, and to compare clinical outcomes and structural integrity between the C-shaped and the established crescent-shaped small to medium-sized rotator cuff tears after arthroscopic repair.

**Methods:**

This retrospective study included 102 patients who underwent arthroscopic repair in a single-row fashion for small- to medium-sized rotator cuff tears of either C or crescent shape from March 2009 to June 2014. Visual analogue scale (VAS) pain score, subjective shoulder value (SSV), American Shoulder and Elbow Surgeon (ASES) score, and active range of motion (ROM) were evaluated for functional outcomes. Postoperative magnetic resonance arthrography (MRA) or computed tomographic arthrography (CTA) was performed 6 months postoperatively to assess structural integrity.

**Results:**

After 2 years of follow-up, both groups showed no significant difference in VAS pain score, functional scores, or ROM, although the C-shaped tear group exhibited significantly inferior outcomes 3 months after surgery. There was no significant difference in the re-tear rate on follow-up MRA and CTA between groups A and B (24.4 vs. 19.7%, respectively; *p* = 0.570). The postoperative stiffness rate was significantly higher in the C-shaped tear group than that in crescent-shaped tear group only at 3-month follow-up point after surgery (26.8 vs. 9.8%, respectively; *p* = 0.024).

**Conclusions:**

Contrary to our hypothesis, there were no significant differences in functional outcomes and structural integrity between C-shaped and crescent-shaped small- to medium-sized tears 2 years after arthroscopic repair. However, C-shaped tears exhibited significantly worse clinical outcomes, including a higher postoperative stiffness rate than crescent-shaped tears in the early postoperative period at the 3-month follow-up point.

## Backgrounds

Determination and classification of rotator cuff tear configuration is critical for making optimal decisions regarding tension minimization during repair and for increasing the chance for better long-term outcomes. Although there have been several classifications based on three-dimensional or geometric characteristics [1, 2], torn rotator cuff tendons should be reduced onto their footprint in the surgical field, and tension should be evaluated to design an accurate repair because rotator cuff tears can be different at surgery than upon initial presentation.

Typically, with crescent-shaped tears, a direct repair without tension is attainable because these tears are mediolaterally (ML) short, compared to their anteroposterior (AP) length. On the other hand, the direct reduction of the tear edge onto the footprint of longitudinal tears, such as V-shaped or U-shaped tears, is not feasible, and is barely feasible when there is significant tension because of the long ML length. Therefore, a margin convergence technique is recommended to lessen tension while repairing.

**Fig. 1:**
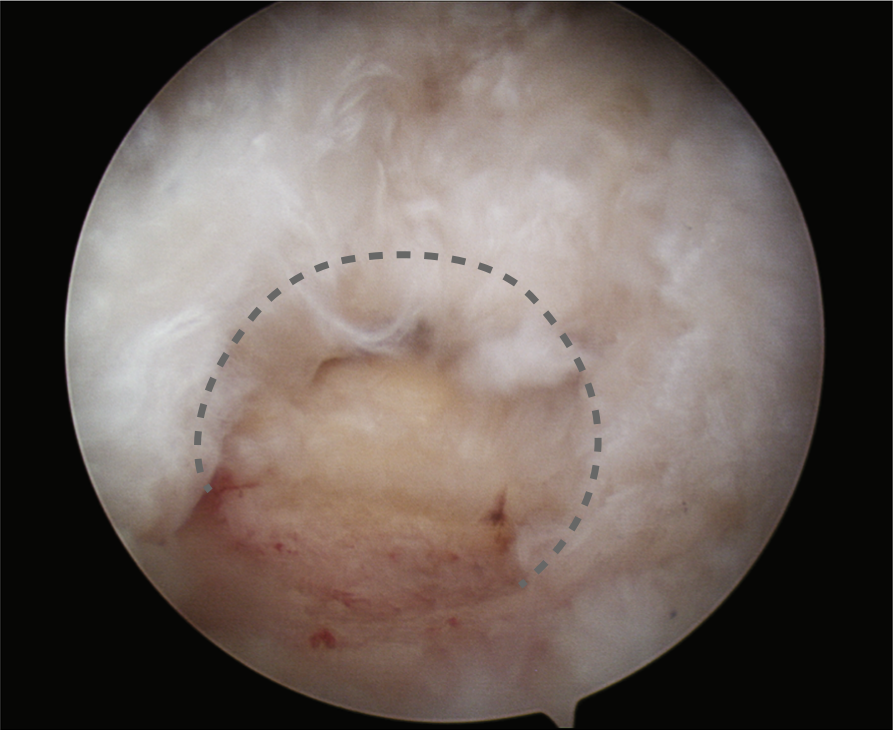
C-shaped tear, Intraoperative arthroscopic finding

**Fig. 2:**
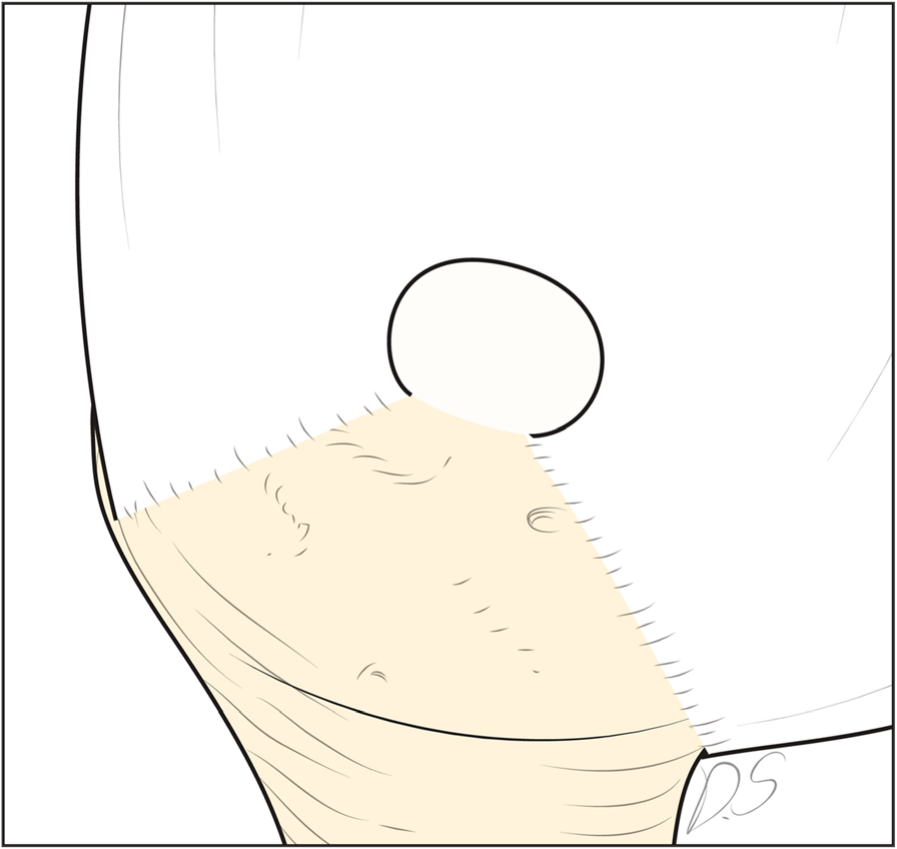
A schematic drawing of C-shaped tear

Although several tear shapes or configurations are recognized in various classification systems, the C-shaped tear is distinctive and does not fit within established rotator cuff tear classifications. C-shaped tears have similar ML and AP length and they are neither crescent-shaped nor U-shaped. Because the size of the tear margin is larger than the footprint, these tears appear like C in Figs. 1 and 2. Because of their unique shape, C-shaped tears are not appropriate for performing margin convergence and may cause a tension mismatch during repair. Therefore, we predicted that the prognosis and clinical outcomes after arthroscopic repair of C-shaped tears would be different from those of similar-sized crescent-shaped tears due to the greater tension during repair.

Herein, we aimed to describe a new type of rotator cuff tear, the C-shaped tear, and to compare clinical outcomes and structural integrity between C-shaped and crescent-shaped small- to medium-sized rotator cuff tears after arthroscopic repair. We hypothesized that C-shaped tears would have inferior clinical outcomes and higher incidence of postoperative stiffness, and that the re-tear rate would be higher for C-shaped tears compared with crescent-shaped tears.

## Methods

### Study population

From March 2009 to June 2014, 131 patients underwent arthroscopic repair in a single-row fashion for small to medium-sized rotator cuff tears with a C or crescent shape: 51 patients had C-shaped tears and 80 patients had crescent-shaped tears. Indication for surgery included pain or functional discomfort during daily-life activities and being refractory to conservative management for at least 3 months. The C-shaped tear group is defined as relatively short and narrow full-thickness supraspinatus tear with or without a concomitant infraspinatus tear with minimal retraction by arthroscopic finding. The medial-to-lateral length of these tears is almost same to anterior-to-posterior width, but the middle part of the medial-to-lateral length, the widest margin of the tear, is greater than the other ends of the medial-to-lateral length looking like C-shape (Figs. 1 and 2). This tear type had a wider tear margin compared to its footprint, causing an inevitable tension mismatch at the repair site. Patients were divided into either a C-shaped tear group (group A) or a crescent-shaped tear group (group B). The inclusion criteria were small to medium-sized tears on MRI or MRA assessment and availability for at least 2 years of follow-up after surgery. Small tears were defined as < 1 cm, and medium tears as 1 to 3 cm [3]. The exclusion criteria were (1) concomitant shoulder stiffness, (2) previous operation history on the affected shoulder, (3) stage IV fatty infiltration in either the subscapularis or the supraspinatus tendon according to Goutallier classification, (4) U-shaped tear requiring margin convergence for complete repair, and (5) having a worker’s compensation claim. In total, 102 patients (41 patients in group A and 61 patients in group B) met the inclusion and exclusion criteria and were, thus, included in this study. The retrospectively reviewed data included medical records and radiologic images. We obtained approval from Institutional Review Board and written informed consent was waived.

### Functional and radiographic assessment

For functional assessments, we used the visual analog scale (VAS) pain score, the subjective shoulder value (SSV), the American Shoulder and Elbow Surgeons (ASES) score, and active range of motion (ROM). (Measurement of ROM included forward flexion in the scapular plane and external rotation with the elbow at the side.) Internal rotation was rated by measuring the highest spinal segment the patient could reach with the thumb pointed upward. To facilitate statistical analyses, spinal column levels were converted to continuous numbers. For example, T1 to T12 were represented by 1 through 12, L1 to L5 were represented by 13 through 17, and the sacrum was represented by 18 [4, 5]. All these values and active ROM were evaluated by an independent examiner who was blinded to preoperative patient data and each postoperative follow-up.

For preoperative radiologic evaluation, true shoulder anteroposterior view, axillary views, and MR images were reviewed. For postoperative radiologic evaluation, postoperative MRA (3.0-T MRA image, Magnetom Tim Trio; Siemens, Erlangen, Germany) or computed tomographic arthrography (CTA) (Somatom Sensation 64; Siemens) was performed 6 months after surgery to assess the structural integrity of the shoulder. Because of the high cost of MRA, 9 out of total 131 patients in this study refused a follow-up MRA. We performed a follow-up CTA alternatively in those patients. We evaluated the structural integrity and classified it according to the French Society of Arthroscopy [6]: stage 1, normal rotator cuff; stage 2, water-tight rotator cuff with intertendinous leakage of dye; stage 3, leakage of dye through a small gap in the rotator cuff; stage 4, leakage of dye through a large gap in the rotator cuff. Stages 1 and 2 were regarded as intact, and stages 3 and 4 were regarded as a re-tear. Additionally, for the patients who had follow-up CTA, we defined re-tear when CTA showed at least minor discontinuity. A single orthopedic surgeon, fellowship trained in shoulder and elbow surgery, did the measurement of the tears.

### Surgical procedure

The arthroscopic repair was performed with the patient in the beach-chair position under general anesthesia. Through the standard posterior portal, the joint was carefully inspected to identify other concomitant intra-articular lesions. If the subscapularis tear needed repair, a simple repair or mattress repair was performed with the suture anchor. In the subacromial space, the lateral viewing portal and anterolateral working portal were established to evaluate the tear configuration, tendon quality, and mobility. The length of the middle part of the medial-to-lateral, the widest margin of the tear was longer than that of the footprint in the C-shaped tear. In this group, over-tensioning during the repair or tension mismatch was inevitable. As the residual tendon tissue was too robust to be sacrificed, we did not take down the residual tissue. In order to reduce the tension while repairing, the anchor was inserted into the medial portion within the footprint. After the footprint and tear margin were prepared, the suture anchor was inserted as necessary. Using a suture passer (Scorpion; Arthrex, Naples, FL, USA), the sutures were passed through the tendon, and the tendon was repaired. In this study, we included only the single row repair cases.

### Postoperative rehabilitation

The affected arm was kept in a brace for 6 weeks after surgery. Pendulum and self-assisted circumduction exercises were begun on the first day after surgery. Self-assisted passive ROM exercises, such as the table sliding/stretching exercise and forward flexion in the supine position, were begun after 6 weeks postoperatively. Self-assisted active ROM exercises were encouraged after 8 weeks postoperatively. 3 months after surgery, isotonic strengthening exercises with an elastic band were started. 6 months after surgery, the patients were allowed to gradually return to their premorbid level of sports activities.

### Statistical analysis

All statistical analyses were performed with SPSS software (version 21.0; IBM, Armonk, NY, USA). The Student’s *t* test was used to compare continuous or continuously ranked data, including VAS score and shoulder scores (SSV and ASES), between the two groups. The paired *t* test was used to compare preoperative and postoperative values within each group. For categorical data, the chi-square test was used to compare between the two groups. Statistical significance was set as *p* < 0.05.

## Results

### Patient demographics

Group A was composed of 15 men and 26 women, and group B was composed of 23 men and 38 women. The mean age at surgery was 61.6 years (range 47–72 years) in group A and 60.4 years (range 43–80 years) in group B. The mean symptom duration before surgery was 19.4 months (range 9–34 months) in group A and 20.3 months (range 9–34 months) in group B. The dominant arm was involved in 29 patients (70.7%) in group A and in 44 patients (72.1%) in group B. There were no significant differences between the two groups (Table 1).

**Table 1:** Patient demographics

	Group A (*n* = 41)	Group B (*n* = 61)	*p* value
Sex, male/female, *n*	15/26	23/38	0.909
Age, mean ± SD, years	61.6 ± 6.7 (47–72)	60.4 ± 7.8 (43–80)	0.406
Symptom period, mean ± SD, months	19.4 ± 5.6 (9–34)	20.3 ± 5.6 (9–34)	0.439
Dominant arm involvement, *n* (%)	29 (70.7)	44 (72.1)	0.878

### Tear size on MR images and arthroscopic concomitant procedures

The mean anterior-to-posterior tear dimension was 16.5 ± 3.9 mm (range 10.2–23.4 mm) in group A and 19.6 ± 5.9 mm (range 10.0–29.1 mm) in group B, and the mean medial-to-lateral tear dimension was 21.9 ± 4.8 mm (range 14.8–29.6 mm) in group A and 13.3 ± 4.4 mm (range 7.1–21.2 mm) in group B. Twelve patients (29.3%) in group A and 19 patients (31.1%) in group B had a concomitant subscapularis tear requiring a repair. A biceps lesion or a SLAP (superior labral anterior-posterior) lesion was found in 16 patients (39.0%) in group A and in 24 patients (39.3%) in group B. All biceps or SLAP lesions were treated with a biceps tenotomy or tenodesis. The mean number of anchors for rotator cuff tendon repair in this study was 2.0 ± 0.8 in group A, and 1.9 ± 0.8 in group B, respectively.

### Functional and radiological assessments

At the 2-year follow-up point, the mean VAS pain score improved from 6.9 ± 1.7 to 1.4 ± 1.3 in group A (*p* < 0.001) and from 7.4 ± 1.9 to 1.1 ± 1.2 in group B (*p* < 0.001). There was no significant difference in postoperative VAS pain score between the two groups. The mean SSV improved from 34.9 ± 15.7 to 89.2 ± 13.8 in group A (*p* < 0.001) and from 31.4 ± 16.2 to 91.1 ± 12.7 in group B (*p* < 0.001) at the 2-year follow-up point, but the postoperative difference was not significant between the groups. The mean ASES score improved from 35.4 ± 7.4 to 88.0 ± 4.7 in group A (*p* < 0.001) and from 36.8 ± 5.3 to 89.4 ± 6.1 in group B (*p* < 0.001) at the 2-year follow-up point; the postoperative difference was not significant between the groups (Table 2). The mean preoperative active forward flexion was 139.3° ± 9.9° in group A and 140.7° ± 9.5° in group B (*p* = 0.511); mean postoperative active forward flexion improved significantly to 151.8° ± 8.4° in group A (*p* < 0.001) and 154.1° ± 7.1° in group B (*p* < 0.001). The mean external rotation improved significantly from 49.0° ± 5.0° to 54.1° ± 4.4° in group A (*p* < 0.001) and from 49.4° ± 4.6° to 54.6° ± 6.1° in group B (*p* < 0.001). The mean internal rotation improved significantly from 13.0 ± 1.8 to 9.9 ± 1.0 in group A (*p* < 0.001) and from 13.3 ± 1.4 to 9.8 ± 1.5 in group B (*p* < 0.001). For all active range-of-motion measurements, preoperative and postoperative differences between the two groups were not significant (Table 3). However, at an early follow-up point (3 months after surgery), the C-shaped tear group had significantly inferior outcomes for VAS pain score and functional scores although there were no significant differences 6 months, 1 year, and 3 years after surgery (Table 2).

**Table 2:** VAS, SSV, and ASES scores in both groups

	Group A (*n* = 41)	Group B (*n* = 61)	*p* value
VAS			
Preoperative	6.9 ± 1.7 (3–10)	7.5 ± 1.9 (3–10)	0.133
3 months follow-up	3.1 ± 1.1 (0–5)	2.6 ± 1.4 (0–5)	0.031^*^
6 months follow-up	1.8 ± 1.2 (0–3)	1.6 ± 1.4 (0–4)	0.390
12 months follow-up	1.6 ± 1.3 (0–4)	1.4 ± 1.2 (0–3)	0.342
24 months follow-up	1.3 ± 1.1 (0–4)	1.2 ± 1.1 (0–4)	0.562
SSV			
Preoperative	34.9 ± 15.7 (0–60)	31.2 ± 16.1 (10–65)	0.259
3 months follow-up	53.7 ± 14.1 (30–90)	60.8 ± 14.0 (40–90)	0.013^*^
6 months follow-up	75.4 ± 13.5 (50–90)	78.0 ± 11.1 (55–90)	0.278
12 months follow-up	83.8 ± 9.0 (50–95)	85.9 ± 7.4 (65–95)	0.198
24 months follow-up	89.2 ± 13.8 (50–100)	91.2 ± 12.6 (50–100)	0.450
ASES score			
Preoperative	35.4 ± 7.4 (16.7–55.0)	36.7 ± 5.3 (30.0–60.0)	0.307
3 months follow-up	61.3 ± 16.4 (45.0–95.0)	68.9 ± 15.7 (45.0–95.0)	0.021^*^
6 months follow-up	87.8 ± 7.7 (70.0–100.0)	89.5 ± 5.6 (75.0–100.0)	0.213
12 months follow-up	89.4 ± 4.0 (80.0–95.0)	90.1 ± 5.1 (76.7–100.0)	0.484
24 months follow-up	88.0 ± 4.7 (71.7–96.7)	90.0 ± 6.2 (71.7–100.0)	0.157

^*^Significant difference, *p* < .05

**Table 3:** Active range of motion in both groups

	Group A (*n* = 41)	Group B (*n* = 61)	*p* value
Forward flexion			
Preoperative	139.3 ± 9.9 (122–162)	140.7 ± 9.5 (120–160)	0.476
3 months follow-up	125.8 ± 16.3 (100–162)	127.2 ± 17.2 (100–160)	0.688
6 months follow-up	133.5 ± 17.3 (110–162)	135.1 ± 18.8 (110–162)	0.651
12 months follow-up	148.9 ± 8.8 (125–165)	149.4 ± 9.9 (127–165)	0.759
24 months follow-up	151.8 ± 8.4 (135–165)	154.1 ± 7.1 (140–165)	0.147
External rotation			
Preoperative	49.0 ± 5.0 (40–58)	49.5 ± 4.7 (40–58)	0.599
3 months follow-up	28.0 ± 5.7 (20–37)	29.0 ± 5.0 (20–40)	0.379
6 months follow-up	43.9 ± 7.2 (27–54)	45.1 ± 11.7 (22–61)	0.555
12 months follow-up	51.9 ± 4.8 (40–60)	51.6 ± 6.2 (35–62)	0.280
24 months follow-up	54.1 ± 4.4 (40–60)	54.6 ± 6.1 (37–65)	0.711
Internal rotation			
Preoperative	13.0 ± 1.8 (11–17)	13.3 ± 1.4 (8–17)	0.293
3 months follow-up	15.3 ± 1.7 (10–17)	15.0 ± 1.7 (11–17)	0.384
6 months follow-up	14.7 ± 1.5 (10–17)	14.6 ± 1.9 (10–17)	0.830
12 months follow-up	11.3 ± 1.8 (7–16)	11.0 ± 1.6 (7–14)	0.377
24 months follow-up	9.9 ± 1.0 (7–12)	9.8 ± 1.5 (7–16)	0.626

We defined postoperative stiffness of the affected shoulder as when passive forward flexion was limited less than 120° or external rotation less than 30° with the arm at the side, or internal rotation less than L-3 at the back [7]. Using these criteria, we estimated 26.8% stiffness prevalence in group A and 9.8% in group B 3 months after surgery, 4.9% stiffness in group A and 3.3% in group B after 6 months, 4.9% in group A and 1.6% in group B after 12 months, and 2.4% in group A and 1.6% in group B at the final follow-up point. The 3-month follow-up was the only point that the postoperative stiffness rate was significantly higher in group A than in group B. For patients who had postoperative stiffness, especially those who had pain interrupting their sleep at night, we administered an ultrasound-guided intra-articular corticosteroid injection. Most postoperative stiffness eventually resolved in both two groups. The remaining stiffness rate was very low in group A and group B, and at the final follow-up there was not a significant difference between the two groups (Table 4).

**Table 4:** Postoperative stiffness rate in both groups

	Group A (*n* = 41)	Group B (*n* = 61)	*p* value
Postoperative stiffness, *n* (%)			
3 months follow-up	26.8 (11)	9.8 (6)	0.024^*^
6 months follow-up	4.9 (2)	3.3 (2)	0.687
12 months follow-up	4.9 (2)	1.6 (1)	0.347
24 months follow-up	2.4 (1)	1.6 (1)	0.778

^*^Significant difference, *p* < .05

Postoperative follow-up MRA or CTA was performed on 36 patients (87.8%) in group A and 53 patients (86.9%) in group B at the 6-month follow-up point. Re-tears were identified in 10 patients (24.4%) in group A and 12 patients (19.7%) in group B. However, there was no significant difference in the re-tear rate between the groups (*p* = 0.570).

## Discussion

Our goal for this study was to describe a new rotator cuff tear pattern, the C-shaped tear, and to compare its clinical outcomes and structural integrity after arthroscopic repair with those for crescent-shaped tears, focusing on small-to-medium sized tears. We predicted that C-shaped tears would yield inferior clinical and radiological outcomes compared with crescent-shaped tears because they were repaired under higher tension conditions compared to Crescent-shaped tears, even though not as high as in U-shaped tears. However, contrary to our hypothesis, there were no significant differences in clinical outcomes or structural integrity between the two surgical groups at the two-year follow-up point. However, in the early postoperative period there were significant differences in the affected shoulder joint for ROM and functional scores.

Many investigators [8–10] indicated that tension mismatch while repairing rotator cuff tears was related to postoperative pain and stiffness. Franceschi et al. [11] reported that patients who underwent partial repair for massive irreparable rotator cuff tears continued to experience a painful shoulder, especially with nocturnal pain for the first post-operative month and they mentioned this may result from the tissue tensioning consequent to the partial repair of the rotator cuff. Shin et al. [9] reported that a tension mismatch between the repaired portion and the adjacent normal portion could occur while repairing a small sized rotator cuff tear because more tension would be loaded in the repaired portion. This would also lead to biomechanical changes in the repaired tendon and resulting postoperative stiffness. The majority of small to medium-sized tears included in our study were single-tendon tears, and we thought that a C-shaped tear configuration yielded a more severe tension mismatch to the adjacent normal tendon portion because it had significantly longer medial-to-lateral length than the crescent-shaped tear configuration. However, we could not quantitatively measure the tension difference in this study. Despite differences in functional outcomes and ROM in the early postoperative period, there were no significant differences at the 2-year follow-up point.

Although stiffness is the most common complication after rotator cuff repair [8, 12], postoperative stiffness has not been clearly defined. Huberty et al. [10] defined stiffness according to patient dissatisfaction, and patients who reported having a disabling lack of shoulder motion were regarded as having a stiff shoulder. On the basis of this definition, they found that 24 of 489 patients (4.9%) developed postoperative shoulder stiffness after rotator cuff repair after an average of 8 months. Brislin et al. [8] defined shoulder stiffness as having < 100° forward flexion, external rotation < 10° with the arm at the side, or external rotation < 30° with the arm in 90° abduction; they found postoperative shoulder stiffness after arthroscopic rotator cuff repair in 23 of 268 patients (8.6%) 3 months after surgery. Recently, Oh et al. [7] defined stiffness as forward flexion < 120°, external rotation < 30° with the arm at the side, or internal rotation at the back of less than L-3. Using these criteria, they found stiffness in 18.6% of patients 3 months after rotator cuff repair, in 2.8% of patients 6 months after repair, and in 6.6% of patients at the final follow-up point. In this study, we found significantly more postoperative stiffness in patients with C-shaped tears during the early postoperative period (3 months of follow-up).

Many studies have reported that factors that contribute to healing failure of a repaired rotator cuff include excessive tension during repair, tear size, and quality of the tendon [13–17]. Among these factors, over-tensioning during the repair can be controlled with proper surgical fixation, tying with appropriate tension, and maintaining proper arm position after operation [18–20]. Davidson et al. [21] suggested that rotator cuff repair tension correlates directly with surgical outcomes; thus, they recommend < 8 lbs of tension during repair. Kim et al. [18] reported that rotator cuff repair tension was inversely correlated with healing at the repair site. When we reduced the tear edge onto the footprint, we felt that it was stiffer in the C-shaped tear configuration, even though we did not employ a device for measuring tension during repair and could not quantify it.

Several studies have evaluated the re-tear rates after arthroscopic rotator cuff repair, and the reports are highly variable. Cho et al. [22] reported on the factors affecting clinical outcomes and integrity after arthroscopic rotator cuff repair, and they found that, after 6 months, the healing rate increased as the tear-size decreased. They reported a 3.3% re-tear rate for small-sized tears, a 12.7% re-tear rate for medium-sized tears, and a 41.2% of re-tear rate for large or massive tears. In contrast, Vavken et al. [23] estimated that the high end of the range of re-tear rates was as much as 30% for small-to-medium tears and up to 94% for large tears. Herein, we found that the re-tear rate after 6 months was 14.6% for group A and 9.8% for group B. We hypothesized that the re-tear rate would be higher for C-shaped tears than for crescent-shaped tears because of the unique C-shaped tear pattern that can cause excessive tension and tension mismatch at the repair site. However, with the exception of re-tear rates, which were higher for C-shaped tears, there was no significant difference between the two groups. We think that the tear sizes we evaluated might explain these results. By only including small-to-medium tears, we might not have been observing cases where excessive tension and tension mismatch actually differed significantly between the tear types.

### Limitation

This study has several limitations. First, we subjectively determined tear configuration to be either C-shaped and crescent-shaped, and this may have been an inherently bias approach. Second, as mentioned above, we were not able to measure tension load during repair. Although we thought that C-shaped tears had higher tension loads than crescent-shaped tears, we cannot verify this assumption. Third, the difference in clinical outcomes after 3 months became insignificant from 6 months. This could be attributable to the corticosteroid injections we administered, which could have introduced another source of bias. On the other hand, this lack of difference in both clinical outcomes and structural integrity might be attributable to a type II error by a low statistical power.

## Conclusion

Contrary to our hypothesis, there were no significant differences in functional outcomes and structural integrity between C-shaped and crescent-shaped small to medium-sized tears after 2 years of follow-up from arthroscopic repair. However, C-shaped tears exhibited significantly worse clinical outcomes than crescent-shaped tears, including greater postoperative stiffness, within 3 months after surgery.

## Abbreviations

ASES: American Shoulder and Elbow Surgeons
CTA: Computed tomographic arthrography
MRA: Magnetic resonance arthrography
ROM: Range of motion
SLAP: Superior labrum anterior to posterior
SSV: Subjective shoulder value
VAS: Visual analog scale
